# Biological Aortic Heart Valve Prosthesis in Patients Under 65 Years Old: Are the Paradigms Changing?

**DOI:** 10.21470/1678-9741-2018-0606

**Published:** 2018

**Authors:** Paulo Roberto B. Evora, Domingo M. Braile

**Affiliations:** 1 Editor-in-Chief Interim – BJCVS Faculdade de Medicina de Ribeirão Preto da Universidade de São Paulo (FMRP-USP), Ribeirão Preto, SP, Brazil; 2 Editor-in-Chief - BJCVS Faculdade de Medicina de São José do Rio Preto (FAMERP), São José do Rio Preto, SP, Brazil and Universidade de Campinas (UNICAMP), Campinas, SP, Brazil

## BJCVS Highlight

For patients aged between 60 and 65 years at the time of surgery, the current guidelines of the major international cardiovascular societies propose that mechanical or biological heart valve prosthesis are acceptable. However, the evidence is still insufficient to recommend biological valves for patients younger than 60 years, except in patients who have significant medical contraindications to anticoagulant therapy. According to Hajj-Chahine et al.^[1]^, it is possible to think about a novel lower age threshold for the use of biological aortic valves. However, even considering the adverse effects of lifetime anticoagulation, new biological valves (less prone to degeneration) and new technologies (for example TAVI), or even observing a trend towards even younger patients in recent years, it is interesting to analyze the results of aortic bioprosthetic valve replacement in patients aged <65 years at the time of surgery. This evidence would lead patients and surgeons to different aortic valve prosthesis choices and guidelines changes in a near future^[2]^.

Otherwise, it it well known today that specific groups of the population, such women during their reproductive age, athletes, inhabitants of socially and medically remote areas and patients with visual or mental problems would be good candidates for an aortic bioprosthesis, despite the fact that they were significantly younger than 65 years. However, excluding these specific conditions, most of the published data have a small number of patients and limited follow-up time to allow ample freedom in choosing aortic valve prostheses. In addition, the increased reports about the low rate of reoperations and the low incidence of neurological events provide cardiac surgeons and patients with greater freedom in the selection of aortic bioprostheses. Also, considering the options for interventions with far less morbidity and mortality, making reoperations attractive, it will not surprise that they chose and will keep choosing biological aortic valves over mechanical ones^[3]^. Therefore, it is interesting to keep analyzing the results of aortic bioprosthetic valve replacement in patients aged <65 years at the time of surgery^[2]^.

## Articles in this Issue

This issue of BJCVS presents a blind peer-reviewed selection of 15 papers that were selected by order of acceptance (10 original papers, 2 review articles, and 3 selected case reports).

**Paulo Roberto B. Évora** ^1^Editor-in-Chief Interim - BJCVS Faculdade de Medicina de
Ribeirão Preto da Universidade de São Paulo (FMRP-USP),
Ribeirão Preto, SP, Brazil**Domingo M. Braile** ^2^Editor-in-Chief - BJCVS Faculdade de Medicina de São
José do Rio Preto (FAMERP), São José do Rio Preto, SP,
Brazil and Universidade de Campinas (UNICAMP), Campinas, SP,
Brazil.
